# Supplementary material for: TBX2 is a neuroblastoma core regulatory circuitry component enhancing MYCN/FOXM1 reactivation of DREAM targets
Source: Nat Commun. 2018 Nov 19;9:4866. doi: 10.1038/s41467-018-06699-9 (PMC6242972; doi:10.1038/s41467-018-06699-9)
Supplement: Supplementary file 1 — Supplementary Information [file 41467_2018_6699_MOESM1_ESM.pdf]

Supplementary Information

TBX2 is a neuroblastoma core regulatory circuitry component enhancing MYCN/FOXM1 reactivation of DREAM targets

Decaesteker et al.

Supplementary Figure 1: TBX2 is a SE marked 17q transcription factor in NB

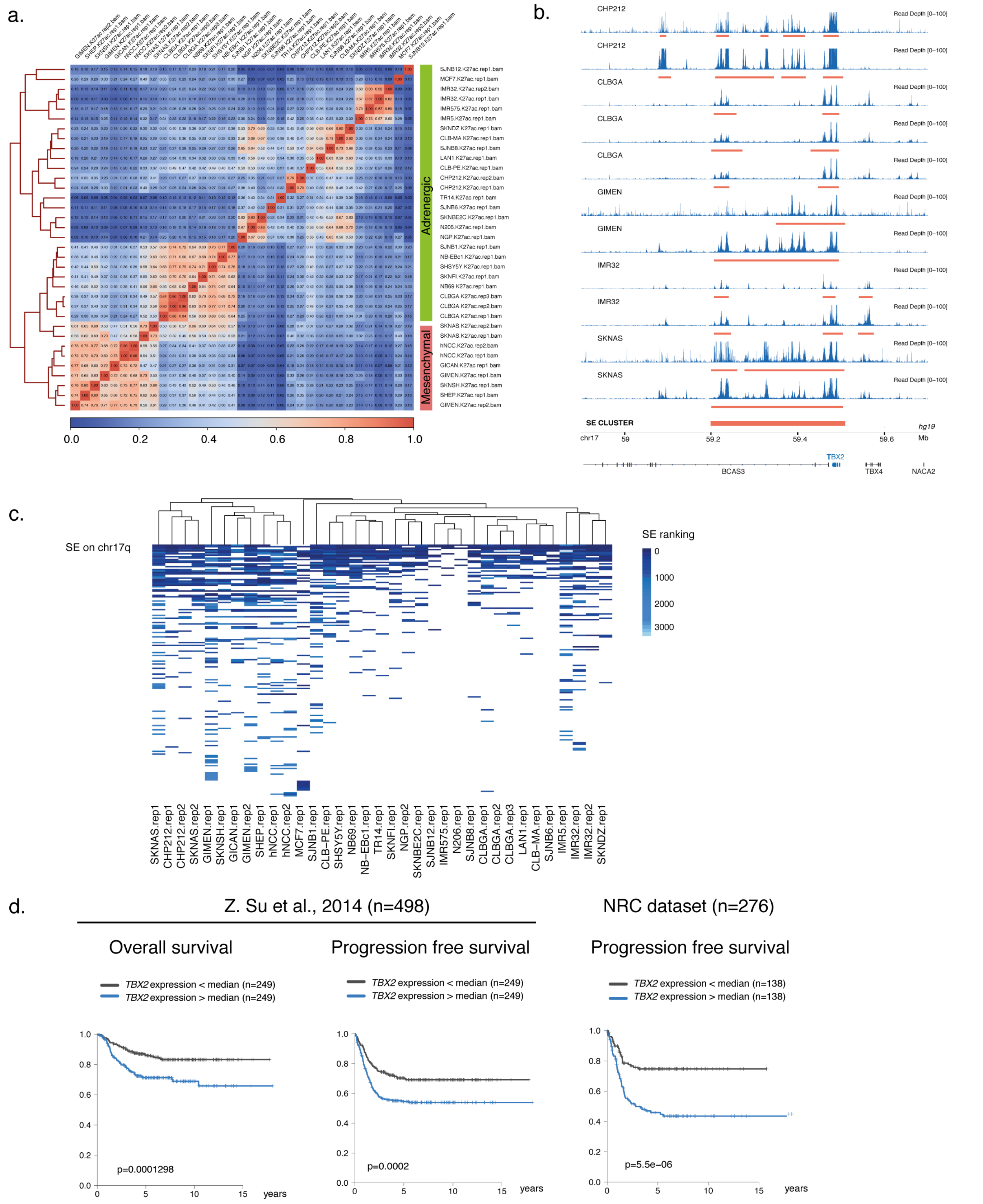

Supplementary Figure 1: *TBX2* is a SE marked 17q transcription factor in NB.
(a). Correlation (Pearson) of H3K27ac profiles in 35 different samples including 26 NB different cell lines, of which 5 are duplicated or triplicated, two non-malignant neural crest cell lines (hNCC) and the non-embryonal breast cancer cell line MCF-7. The correlation of the biological replicates is high for each cell line and cell lines cluster according to adrenergic/mesenchymal subtype[1,2]. **(b).** H3K27ac profiles upstream of *TBX2* in duplicates or triplicates from 6 NB cell lines presented in Fig. 1b. The Lilly annotated SE region is shown in red. The cluster (out of 276 clusters on chr17q) containing the overlapping SEs used in the prioritization process is annotated at the bottom. **(c).** Clusters of SEs on chr17q over 35 different samples including 26 NB different cell lines, of which 5 are duplicated or triplicated, two non-malignant neural crest cell lines (hNCC) and the non-embryonal cell line MCF-7. Blue color intensity indicates the ranking of this SE in the particular cell line according to all other SEs in this cell line. Low value (dark color) means high ranking. **(d).** Kaplan-Meier analysis for overall and progression free survival of 498 neuroblastoma patients (GSE62564) and 276 neuroblastoma patients (GSE85047) with high or low expression (using median as cut-off) of the *TBX2* candidate oncogene.

Supplementary Figure 2: TBX2 is targeted by rare focal amplifications and marked by a SE

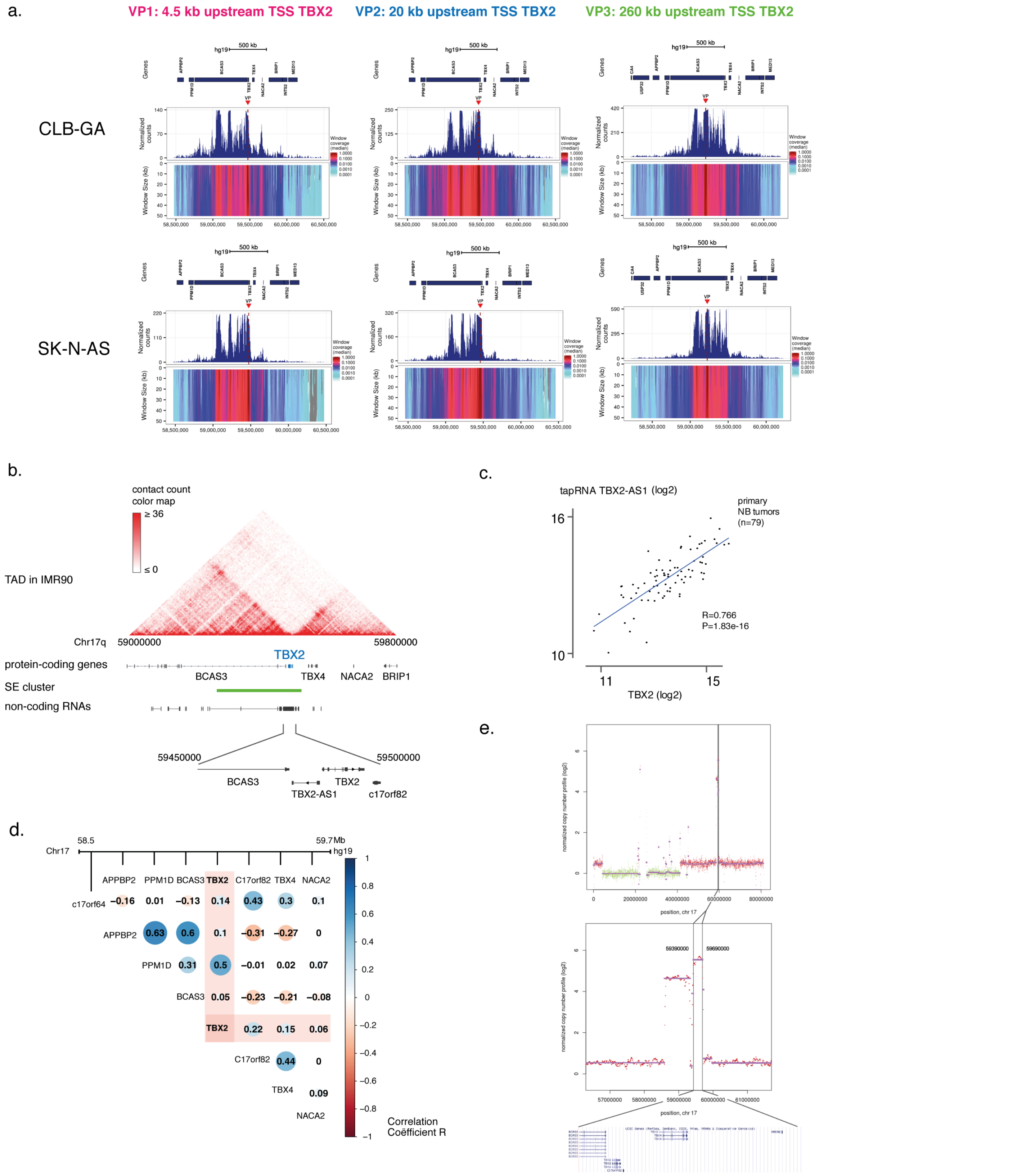

Supplementary Figure 2: ***TBX2*** **is targeted by rare focal amplifications and marked by a SE.**
(a). Reciprocal 4C-seq analysis with three different viewpoints (4.5 kb, 20 kb, 260 kb upstream of *TBX2* TSS) in two different cell lines SK-N-AS and CLB-GA showing interaction between the three hubs in the SE region (promotor site, 260 kb upstream of *TBX2* TSS and 400 kb upstream of *TBX2* TSS). **(b).** *TBX2* is located at the border of a topology associated domain in the IMR-90 cell line measured by Hi-C[3]. Protein coding genes and non-coding genes are depicted underneath the heat map. The tapRNA *TBX2-AS1* is bidirectionally transcribed with *TBX2*. Color range indicates contact count (Image modified from the 3D genome browser: http://www.3dgenome.org). **(c).** *TBX2* mRNA expression is strongly positively correlated with the *TBX2-AS1* lncRNA expression, a highly conserved tapRNA, in primary NB tumors (n=79, p=4.37e-07, R=53 (Spearman correlation)). **(d).** Correlation matrix depicting the correlation of *TBX2* expression with the expression of genes located 1 Mb upstream (including 3 genes) and 0.25 Mb downstream (including 3 genes) of *TBX2*. The size of the dots and the color represent the correlation coefficient (Spearman correlation). **(e).** Log2 copy number ratio of a region on 17q with a focal amplification encompassing the protein-coding genes *BCAS3*, *TBX2*, *C17orf82*, *TBX4* and *NACA2*, as determined by low coverage whole genome sequencing, in a primary NB tumor case (Fig. 1d) (59,390 Mb - 59,690 Mb - hg19 - log2 ratio).

Supplementary Figure 3: TBX2 is involved in a core-regulatory circuitry

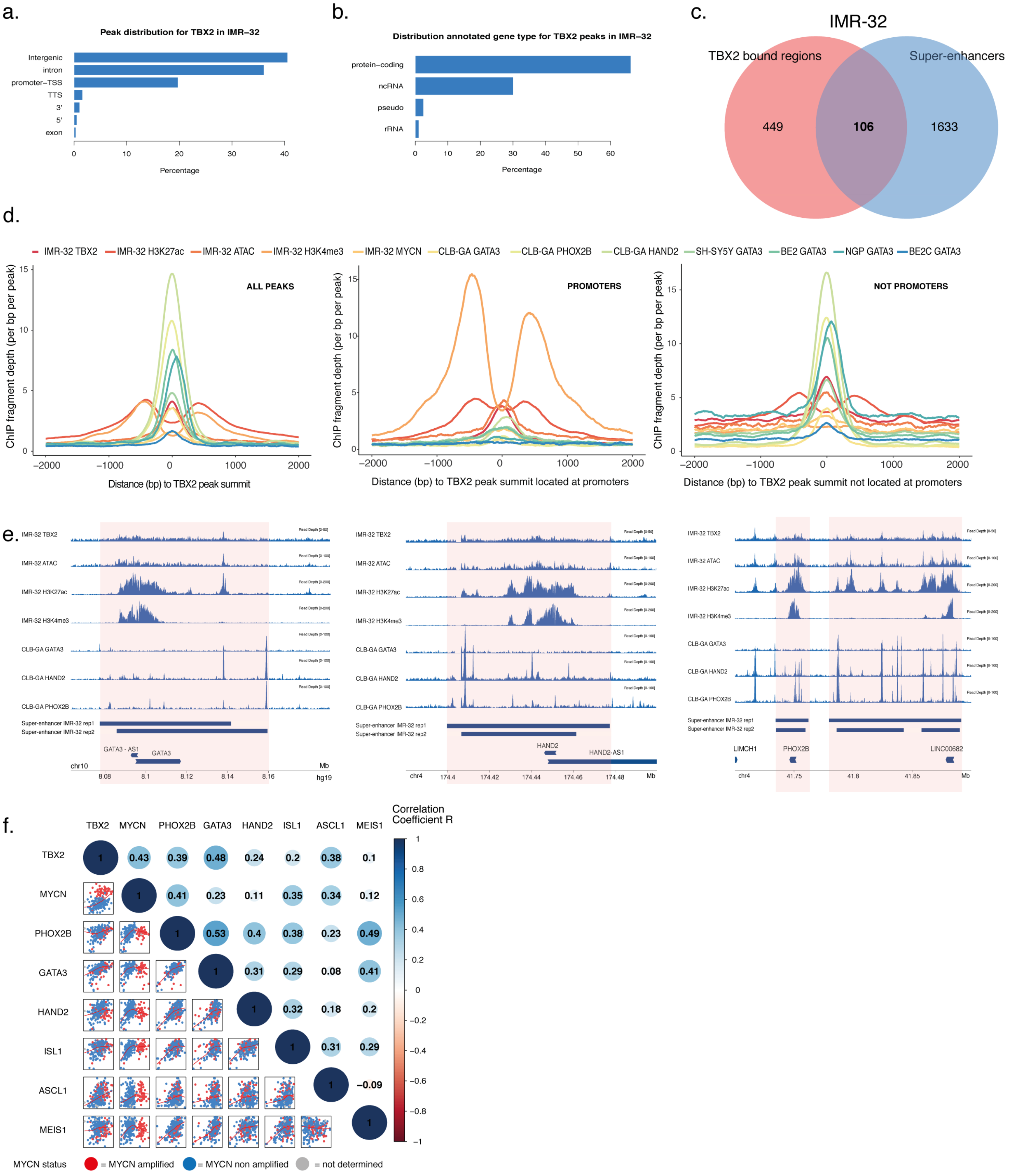

Supplementary Figure 3: ***TBX2*** **is part of a core-regulatory circuitry**

(a). Genomic distribution (%) of TBX2 ChIP-seq peaks in the IMR-32 cell line. **(b).** Functional distribution (%) of the annotated genes (homer annotation) to the TBX2 ChIP-seq peaks in the IMR-32 cell line. **(c).** Overlap (min. overlap = 20 bp) of the TBX2 (qval <0.05) ChIP-seq peaks with the annotated SE in the IMR-32 cell line (Fisher test, p<2.2e-16). **(d).** ChIP fragment depth (per bp per peak) for TBX2, H3K27ac, ATAC, H3K4me3 and MYCN peaks in the IMR-32 cell line, the CRC members GATA3, PHOX2B and HAND2 in the CLB-GA cell line, and GATA3 in the SH-SY5Y, BE2, NGP and BE2C cell lines for the regions -2kb and +2kb from the TBX2 peak summits for all TBX2 peaks (left), those located at promoters (middle) or those not located at promoters (right) (homer annotation). **(e).** ChIP profile of TBX2, ATAC, H3K27ac and H3K4me3 in the IMR-32 cell line, and GATA3, PHOX2B and HAND2 in the CLB-GA cell line, for the three regions around GATA3, HAND2 and PHOX2B. In red, the macs2 called peaks (FDR <0.05) are annotated and below the Lilly called SEs in the two replicates in IMR-32 are depicted. **(f).** Correlation (Spearman) of TBX2 expression with expression of MYCN, PHOX2B, GATA3, HAND2, ISL1, ASCL1 and MEIS1 in the NRC tumor cohort (n=283, GSE85047). The size of the dots and the color represent the correlation coefficient.

Supplementary Figure 4: TBX2 controls a FOXM1/E2F gene regulatory network

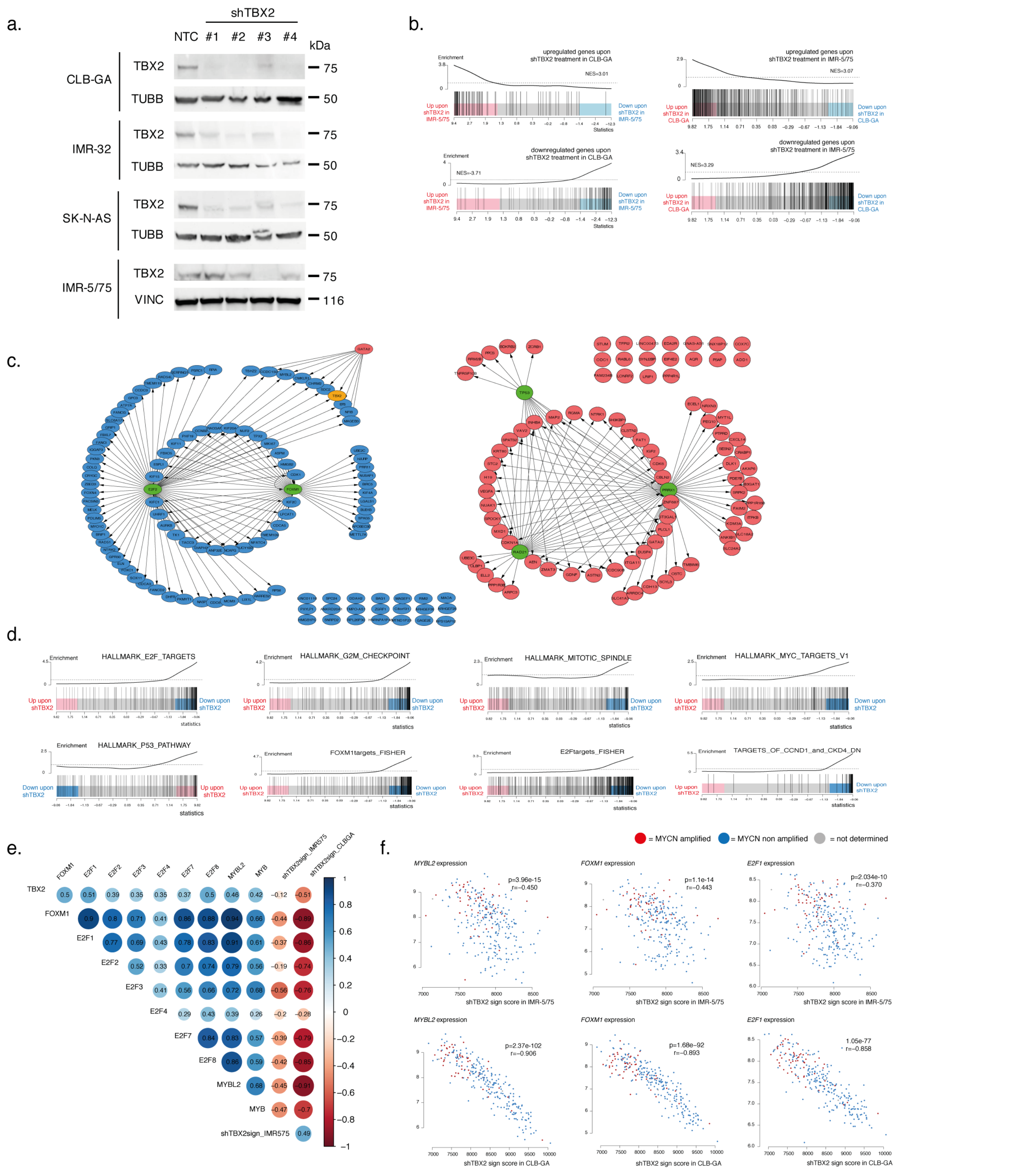

Supplemental Figure 4: TBX2 controls a FOXM1/E2F gene regulatory network.
(a). Reduction of TBX2 protein levels in the CLB-GA, IMR-32, SK-N-AS and IMR-5/75 cell lines upon *TBX2* knockdown with 4 different shRNAs as compared to the non-targeting control (NTC). **(b).** Barcode plots for the up and down sh*TBX2* signatures in CLB-GA and IMR-5/75 mapped on the sh*TBX2* IMR-5/75 (left) and CLB-GA sh*TBX2* data (right). **(c)**. iRegulon motif search for the downregulated (blue nodes, FDR <0.05) and upregulated (red nodes, FDR <0.05) genes upon TBX2 knockdown in CLB-GA. TBX2 is indicated in orange. Genes connected to a gene in the black circles do have a motif enrichment or ChIP-seq binding for the respective gene in that circle. **(d).** Enriched genesets of the MsigDB Hallmarks genesets and genesets from literature (*FOXM1* and *E2F* targets, and the downregulated genes upon *CCND1* and *CDK4* knockdown) for the downwregulated genes upon sh*TBX2* in CLB-GA (FDR <0.01) and the top ranked geneset for the upregulated genes upon sh*TBX2* in CLB-GA (P53 pathway, FDR <0.01). **(e).** Positive correlation between mRNA levels of *TBX2* and other members of the Dream complex and negative correlation with the two different sh*TBX2* signature scores (obtained in CLB-GA and IMR-5/75) in the NRC tumor dataset (n=283, GSE85047). The size of the dots and the color represent the correlation coefficient (Spearman). All correlations are significant (pval <0.05). **(f).** Correlation of *MYBL2*, *FOXM1* and *E2F1* expression as compared to the sh*TBX2* signature score in IMR-5/75 (up) and CLB-GA (down). Color of the dots indicates the *MYCN* status.

Supplementary Figure 5: TBX2 is a cell dependency gene in neuroblastoma cells

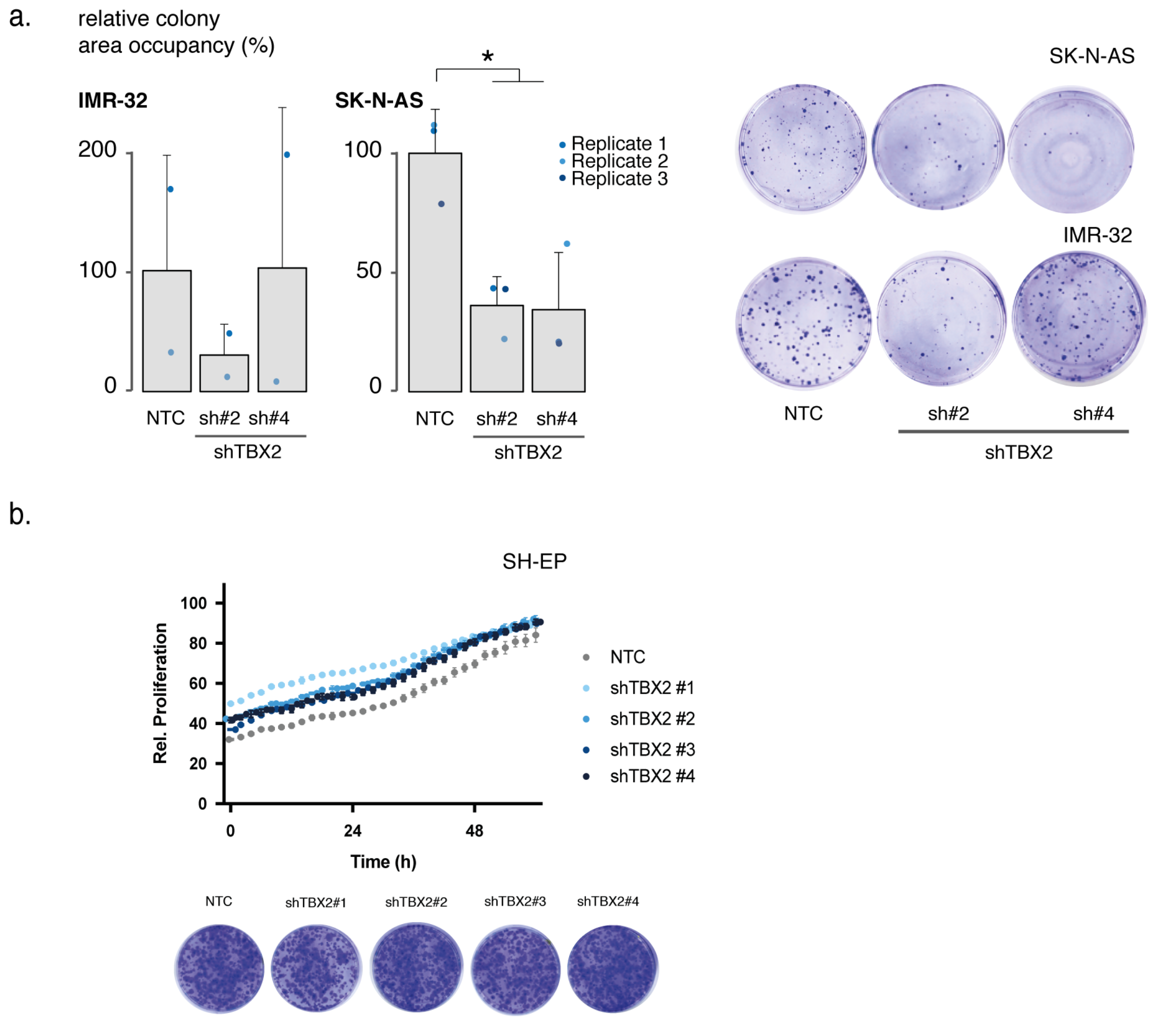

Supplementary Figure 5: TBX2 is a cell dependency gene in neuroblastoma cells
(a). Decrease of %area of colonies in IMR-32 and SK-N-AS cells upon knockdown of *TBX2* as compared to the non-targeting control (NTC). Data-points were mean-centered and error bars represent s.d. of three biological replicates for every cell line. Mann-Whitney test *p<0.05, **p<0.01, ***p<0.001
(b). No reduction of proliferation or colony formation capacity for the SH-EP cell line, which shows that the possible effect of off-targets is limited as *TBX2* is not expressed in SH-EP.

Supplementary Figure 6: Combined TBX2-MYCN signaling targets the FOXM1/E2F network

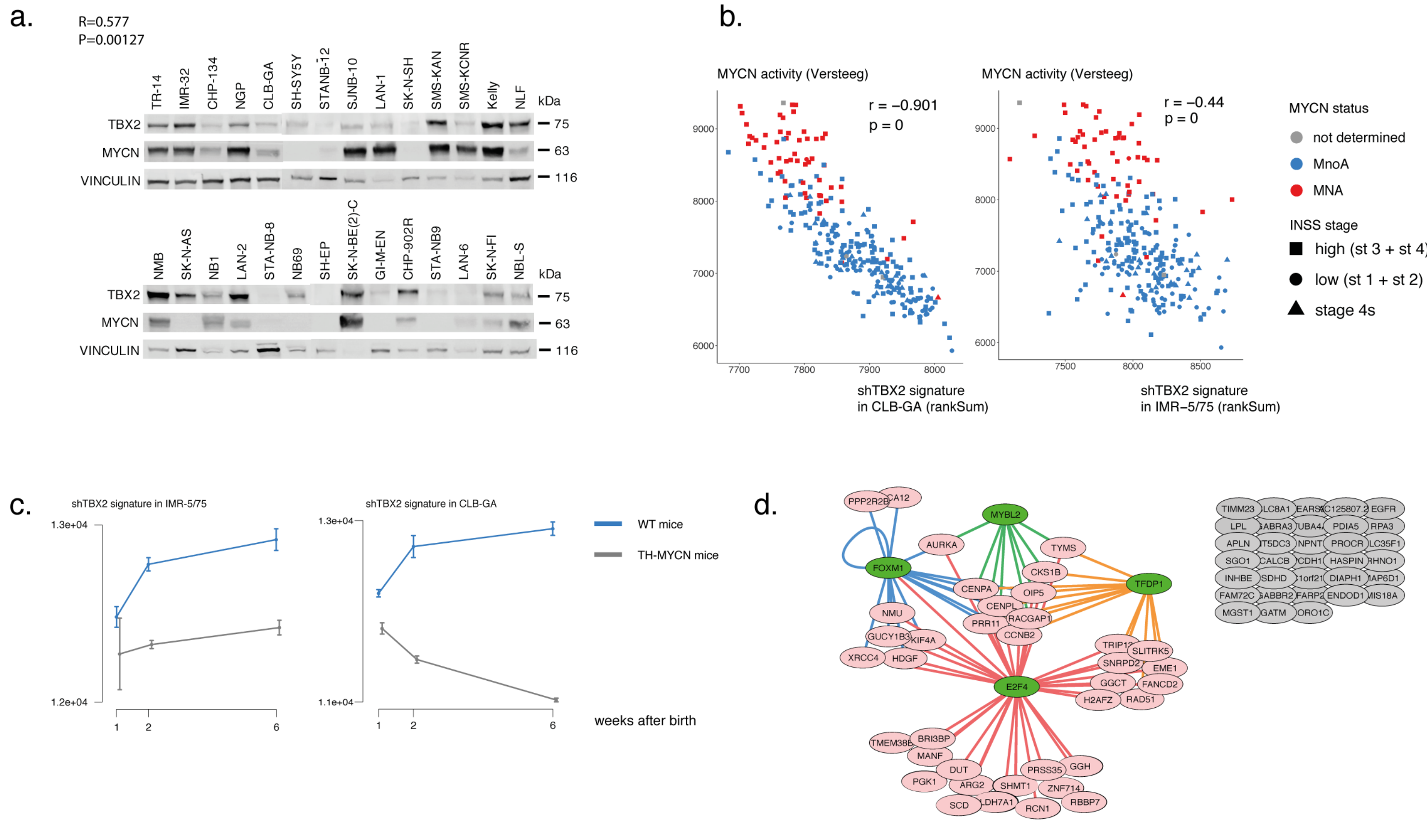

Supplementary Figure 6: Combined TBX2-MYCN signaling targets the FOXM1/E2F network
(a). MYCN and TBX2 protein levels in a panel of 29 neuroblastoma cell lines, quantified with ImageJ, and significantly positively correlated (Spearman correlation), as depicted on the plot (left corner). **(b).** Significant negative correlation (Spearman) of MYCN activity signature score (rankSum) with the sh*TBX2* signature score in CLB-GA (left) or IMR-5/75 (right) (rankSum) in the NRC tumor cohort (n=283, GSE85047). **(c).** sh*TBX2* differential expression analysis signature scores (rankSum) in IMR-5/75 and CLB-GA enriched in normal sympathetic ganglia from wild-type (WT) mice compared to sympathetic ganglia containing hyperplastic lesions and advanced tumors from TH-MYCN mice at respectively, one, two and six weeks after birth. Error bars represent s.d. of four mice per condition. **(d).** Enrichment of motifs for E2F-Dream complex core members in the additively or synergistically downregulated genes upon sh*TBX2*:sh*MYCN* KD.

Supplementary Figure 7: Combined CDK7-BET inhibition as a novel therapeutic approach

a.

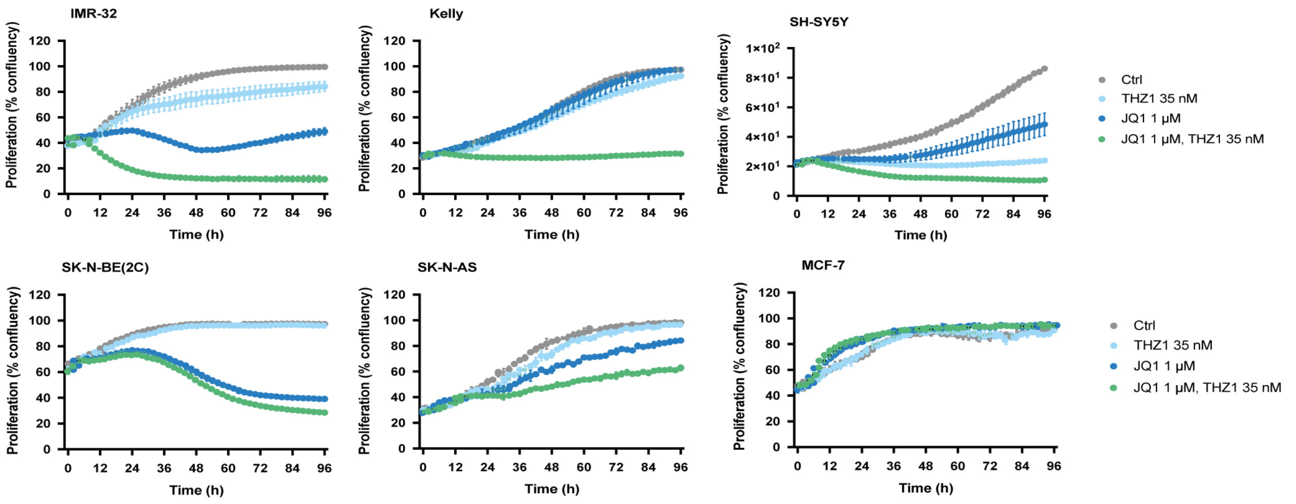

b.

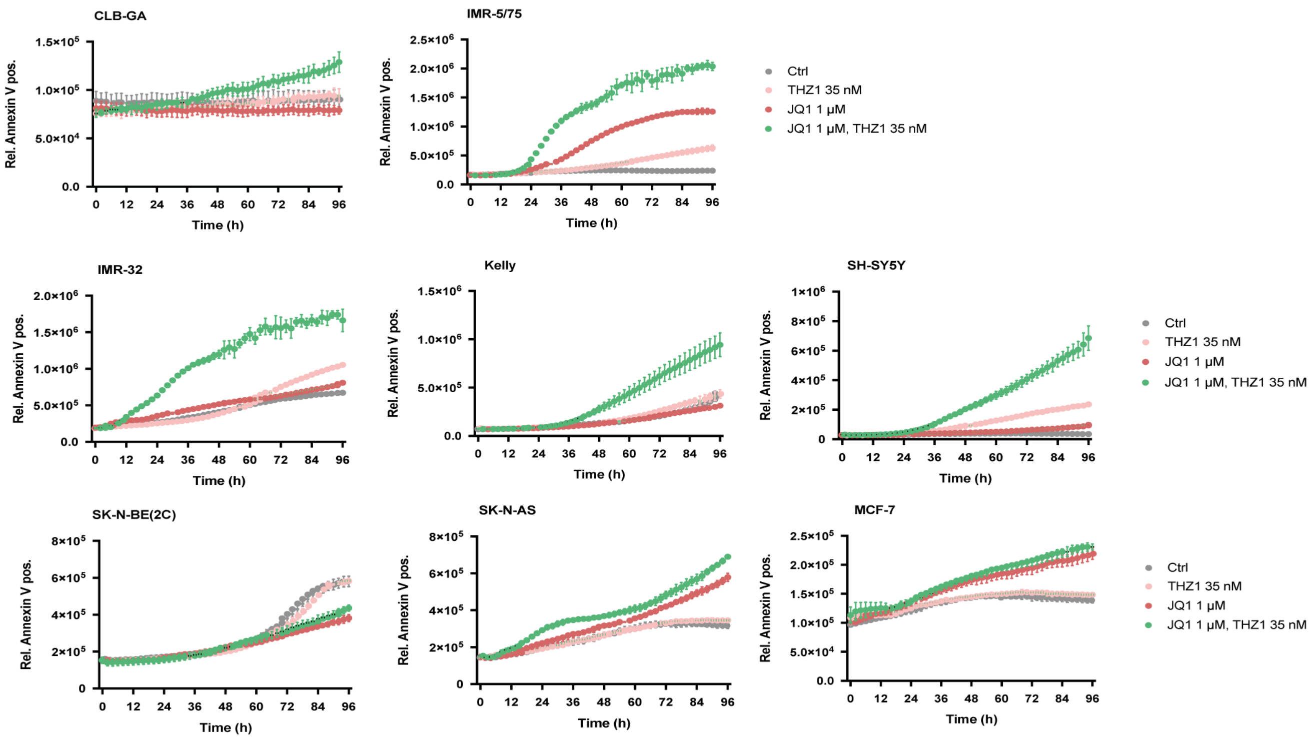

c.

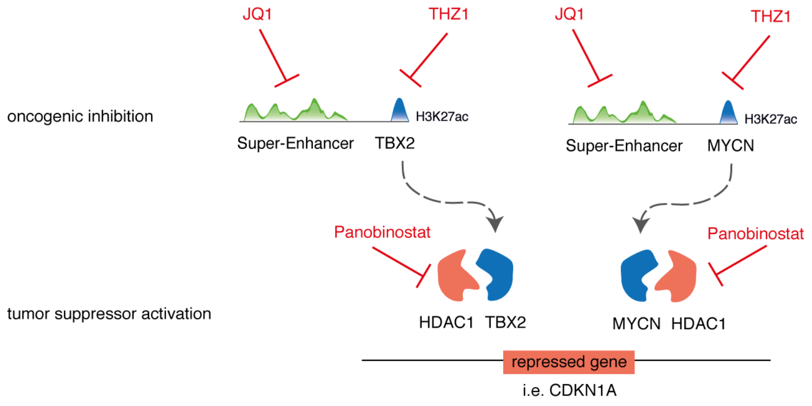

d.

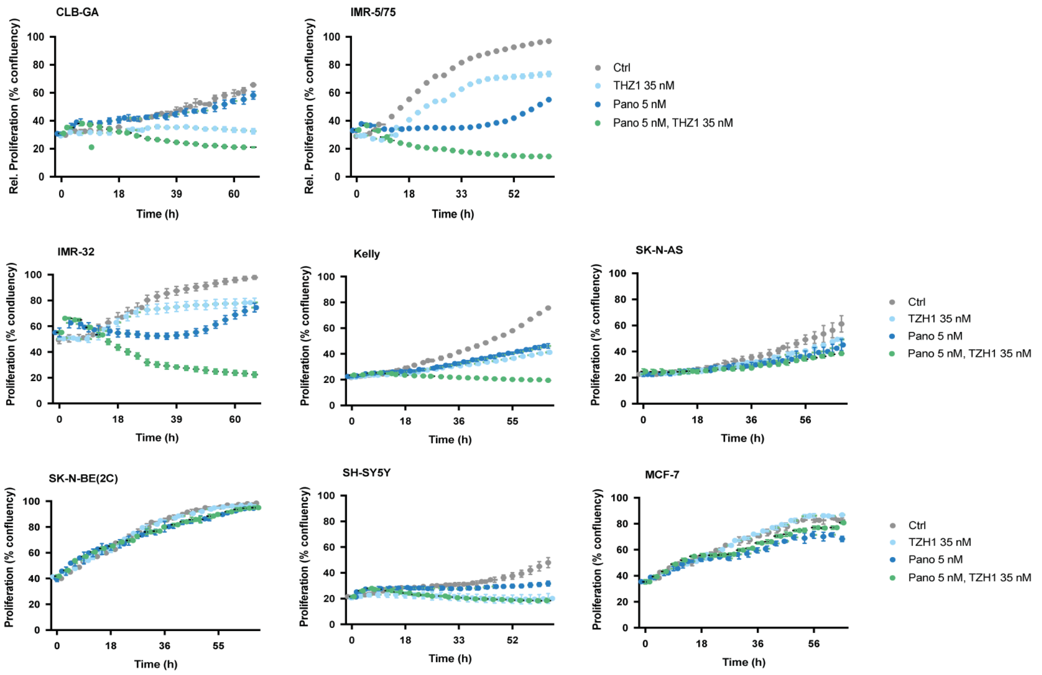

Time (h)	Excess over Bliss CLBGA	IMR-5/75	IMR-32	Kelly	SK-N-AS	SK-N-BE(2C)	SH-SY5Y	MCF7
24	0.158	-0.015	0.271	0.026	-0.026	0.082	0.033	0.179
48	0.168	0.080	0.250	0.098	-0.040	0.038	-0.027	0.063
68	0.101	0.152	0.380	0.078	-0.055	-0.012	-0.064	-0.045

e.

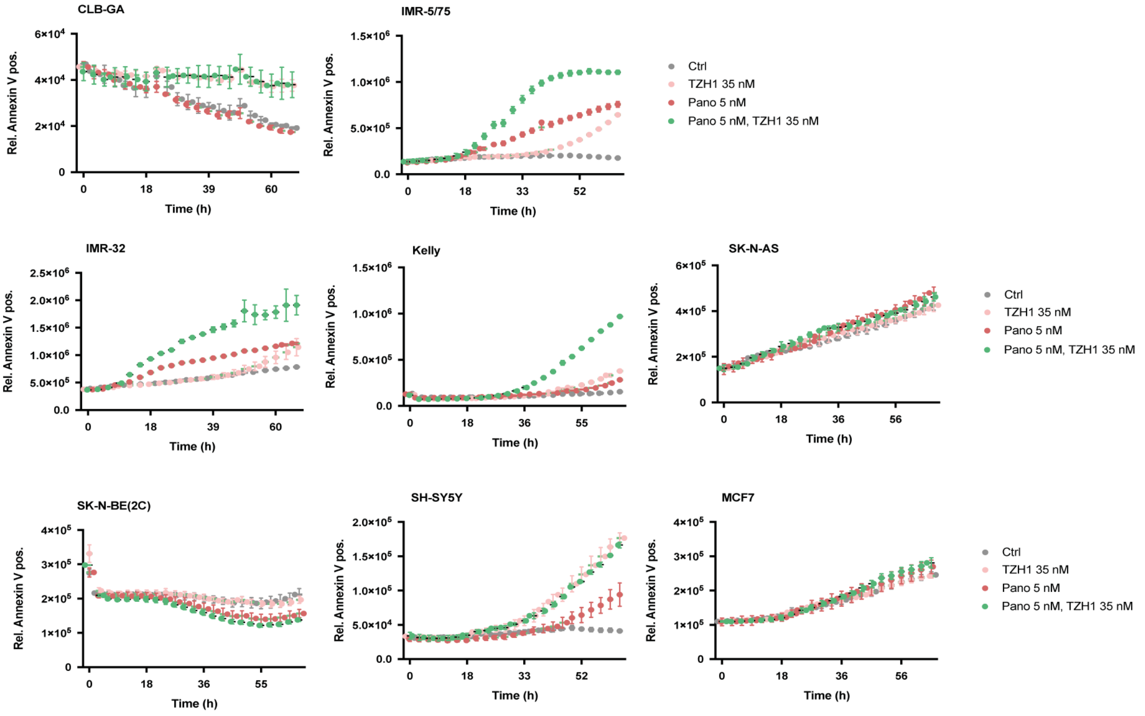

Supplementary Figure 7: Combined CDK7-BET inhibition as a novel therapeutic approach.
(a). Proliferation (%confluency) over time for the cell lines IMR-32, Kelly, SH-SY5Y, SK-N-BE(2C), SK-N-AS and MCF-7 upon treatment with 1 μM JQ1 and 35 nM THZ1 (1 biological replicate out of 3 is shown, error bars represent s.d. of 3 technical replicates)
(b). Relative (Annexin V / % Confluency) AnnexinV staining over time for the cell lines CLB-GA, IMR-5/75, IMR-32, Kelly, SH-SY5Y, SK-N-BE(2C), SK-N-AS and MCF-7 upon treatment with 1 μM JQ1 and 35 nM THZ1. (1 biological replicate out of 3 is shown, error bars represent s.d. of 3 technical replicates). **(c).** Summary scheme to target the CRC affected genes, by treatment with JQ1, THZ1, Panobinostat or the combinations.**(d).** Proliferation (% confluency) over time for the cell lines CLB-GA, IMR-5/75, IMR-32, Kelly, SH-SY5Y, SK-N-BE(2C), SK-N-AS and MCF-7 upon treatment with 35 nM THZ1 and 5 nM Panobinostat. (1 biological replicate out of 3 is shown, error bars represent s.d. of 3 technical replicates). **(e).** Relative (Annexin V / % Confluency) AnnexinV staining over time for the cell lines CLB-GA, IMR-5/75, IMR-32, Kelly, SH-SY5Y, SK-N-BE(2C), SK-N-AS and MCF-7 upon treatment with 35 nM THZ1 and 5 nM Panobinostat (1 biological replicate out of 3 is shown, error bars represent s.d. of 3 technical replicates)

Supplementary Fig 8: Downregulation of the CRC upon combined CDK7-BET inhibition

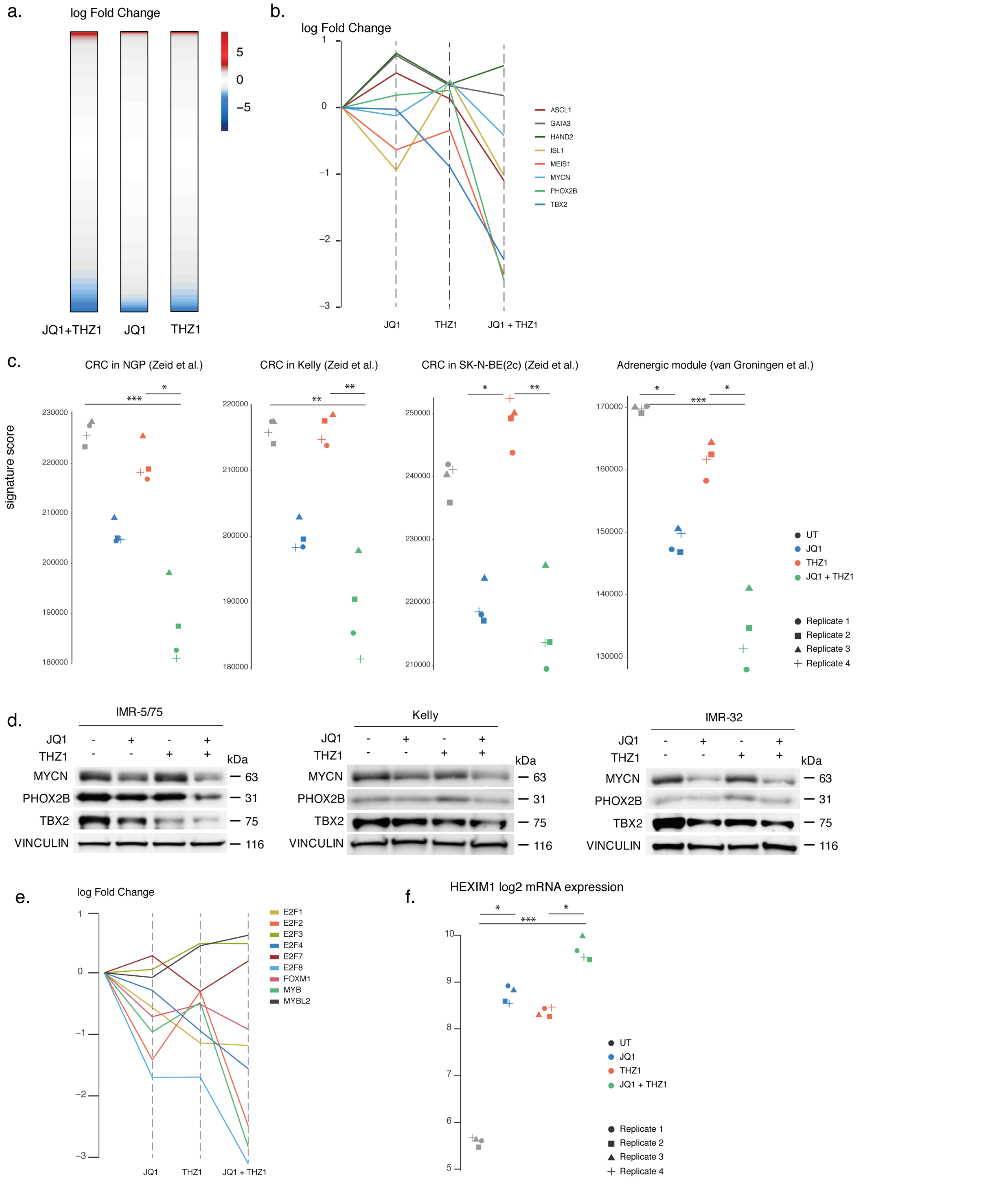

Supplementary Figure 8: Downregulation of the core-regulatory circuitry upon combined CKD7-BET inhibition.
(a). Global gene expression levels upon treatment with 1 μM JQ1, 35 nM THZ1, and the combination in the IMR-5/75 cell line for 10 h, for every comparison represented by the log fold changes of expression (Combination vs control, JQ1 vs control, THZ1 vs control, 4 biological replicates per condition). **(b).** Line plot showing the log fold changes relative to the control for a set of CRC genes upon treatment with 1 μM JQ1, 35 nM THZ1 and the combination in the IMR-5/75 cell line for 10 h (4 biological replicates per condition). **(c).** Significant downregulation of the CRC geneset in NGP, Kelly and SK-N-BE(2c)[4] and adrenergic geneset[1] upon treatment with 1 μM JQ1, 35 nM THZ1 and the combination in the IMR-5/75 cell line for 10 h. (4 biological replicates per condition). **(d).** TBX2, PHOX2B and MYCN protein levels 10 h upon treatment with 1 μM JQ1, 35 nM THZ1 and the combination in the IMR-5/75, Kelly and IMR-32 cell line. **(e).** Line plot showing the log fold changes normalized to the control for the E2F-Dream complex core members upon treatment with 1 μM JQ1, 35 nM THZ1 and the combination of JQ1 and THZ1 in the IMR-5/75 cell line for 10 h. (4 biological replicates per condition). **(f).** *HEXIM1* log2 mRNA expression upon treatment with 1 μM JQ1, 35 nM THZ1 and the combination in the IMR-5/75 cell line for 10 h. (4 biological replicates per condition) Kruskall-Wallis followed by a po¬¬¬st-hoc Dunn's multiple comparisons test: *$p<0.05$, **$p<0.01$, ***$p<0.001$

Supplementary Figure 9
The full blots, where portions of blots and gels have been presented in the main paper

For Fig. 4d

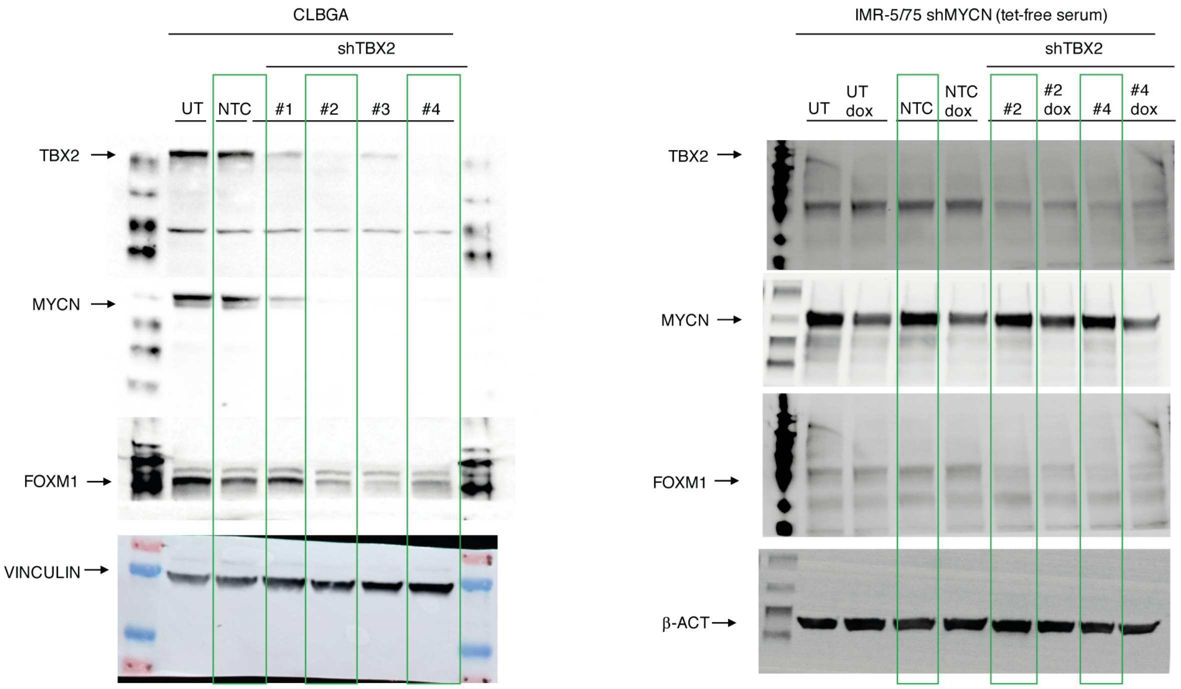

For Fig. 8c

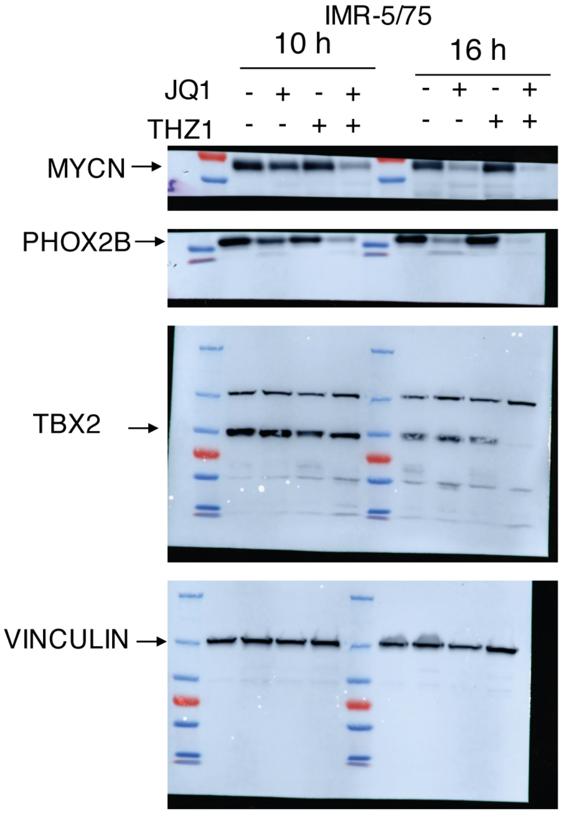

Supplementary Table 1:

Cell lines used in the manuscript with the sample ID, origin, ALK mutation and MYCN amplification status.

Sample ID	Origin	ALK mutation[a]	MYCN status[b]
CLB-GA	Combaret	R1275Q	NA
IMR-32	Versteeg	wt	A
SK-N-AS	ATCC	wt	NA
IMR-5/75	Westermann	wt	A
SH-SY5Y	Schulte	F1174L	NA
SK-N-SH	Versteeg	F1174L	NA
SK-N-FI	Versteeg	wt	NA
SK-N-BE(2c)	Lunec	wt	A
NGP	Versteeg	wt	A
SH-EP	Helen	F1174L	NA
GI-M-EN	Versteeg	wt	NA
CHP-902R	White	wt	A
STA-NB-9	Ambros	wt	A
LA-N-6	White	D1091N	NA
NBL-S	White	wt	NA
Kelly	Luca Lungo Vaschetto	F1174L	A
STA-NB-12	Ambros	wt	NA
SJNB-10	Versteeg	wt	A
LA-N-1	Versteeg	F1174L	A
SMS-KAN	White	Wt	A
SMS-KCNR	Versteeg	F1174L	A
NLF	White	Wt	A
NMB	White	Wt	A
LA-N-2	Versteeg	Wt	A
STA-N-B8	Ambros	F1174L	A
NB-69	Schramm	Wt	NA
TR-14	Versteeg	Wt	A
NB-1	JHSF	wt (ampl)	A
CHP-134	White	Wt	A

[a]wt = wild type
[b]NA = non amplified, A = amplified

Supplementary Table 2:

Sequences of the primers used in the manuscript

Gene	Primer	Sequence
SDHA	Forward	TGGGAACAAGAGGGCATCTG
SDHA	Reverse	CCACCACTGCATCAAATTCATG
HPRT1	Forward	TGACACTGGCAAAACAATGCA
HPRT1	Reverse	GGTCCTTTTCACCAGCAAGCT
TBP	Forward	CACGAACCACGGCACTGATT
TBP	Reverse	TTTTCTTGCTGCCAGTCTGGAC
YWHAZ	Forward	ACTTTTGGTACATTGTGGCTTCAA
YWHAZ	Reverse	CCGCCAGGACAAACCAGTAT
B2M	Forward	TGCTGTCTCCATGTTTGATGTATCT
B2M	Reverse	TCTCTGCTCCCCACCTCTAAGT
TBX2	Forward	AGTGGATGGCTAAGCCTGTG
TBX2	Reverse	ACGGGTTGTTGTCGATCTTC
MYCN	Forward	AGGACACCCTGAGCGATTC
MYCN	Reverse	AGGCATCGTTTGAGGATCAG
PHOX2B	Forward	TAACTGGCTGCAGAGAAATC
PHOX2B	Reverse	GTTCGGATCATTCCAACAGA
LIN28B	Forward	ATAGCACCAGAAGAGCAAAG
LIN28B	Reverse	CCGGGTAAAGGAAAGAACAT
FOXM1	Forward	AGACACCCATTAAGGAAACG
FOXM1	Reverse	TTTGTACTGGGCTGAAATCC
4C TBX2 4,5kB up TSS	Forward	AATGATACGGCGACCACCGAACACTCTTTCCCTACACGACG CTCTTCCGATCTCCTCAAGTCACTCAGTCGATC
4C TBX2 4,5kB up TSS	Reverse	CAAGCAGAAGACGGCATACGAGCCTGCTTCTGCTCTGTC
4C TBX2 SE 20kb up TSS	Forward	AATGATACGGCGACCACCGAACACTCTTTCCCTACACG ACGCTCTTCCGATCTCTCCCTTCCCACTGGAGGATC
4C TBX2 SE 20kb up	Reverse	CAAGCAGAAGACGGCATACGAAACAGTCCTGAGAGACATGG
4C TBX2 SE 260kb up TSS	Forward	AATGATACGGCGACCACCGAACACTCTTTCCCTACACGA CGCTCTTCCGATCTCTTGCTGAATTGAGGTTGATC
4C TBX2 SE 260kb up TSS	Reverse	CAAGCAGAAGACGGCATACGAGCAGAAACACTCCTAAGAGC

Supplementary References:

1. van Groningen, T. *et al.* Neuroblastoma is composed of two super-enhancer-associated differentiation states. *Nat Genet* **49,** ng.3899–1266 (2017).
2. Boeva, V. *et al.* Heterogeneity of neuroblastoma cell identity defined by transcriptional circuitries. *Nat Genet* **49,** ng.3921–1413 (2017).
3. Dixon, J. R. *et al.* Chromatin architecture reorganization during stem cell differentiation. *Nature* **518,** 331–336 (2015).
4. Zeid, R. *et al.* Enhancer invasion shapes MYCN-dependent transcriptional amplification in neuroblastoma. *Nat Genet* **463,** 1 (2018).
