# Supplementary material for: TBX2 is a neuroblastoma core regulatory circuitry component enhancing MYCN/FOXM1 reactivation of DREAM targets
Source: Nat Commun. 2018 Nov 19;9:4866. doi: 10.1038/s41467-018-06699-9 (PMC6242972; doi:10.1038/s41467-018-06699-9)
Supplement: Supplementary file 3 — Description of Additional Supplementary Files [file 41467_2018_6699_MOESM3_ESM.pdf]

## Description of Additional Supplementary Files

File Name: Supplementary Data 1
Description: De novo and known homer motif enrichment (p.val < 1e-10) in the TBX2 bound regions in IMR-32 cells.

File Name: Supplementary Data 2
Description: Significantly up- and down-regulated genes (adj.P.Val<0.05) upon TBX2 knockdown in the IMR-5/75 cell line.

File Name: Supplementary Data 3
Description: Enriched genesets (MsigDB Hallmarks (FDR < 0.05), C5.bp (FDR < 0.01), C2 (FDR < 0.05 with max top 50)) in the down- and up-regulated genes upon shTBX2 KD in CLB-GA and IMR-5/75.

File Name: Supplementary Data 4
Description: iRegulon enrichment table for up- and down-regulated genes upon TBX2 KD in CLB-GA and IMR-5/75, and the additively or synergistically up- and down-regulated genes upon TBX2 and MYCN KD in IMR-5/75.

File Name: Supplementary Data 5
Description: Significantly up- and down-regulated genes (adj.P.Val < 0.05) upon TBX2 knockdown in the CLB-GA cell line.

File Name: Supplementary Data 6
Description: Significantly synergistically up- and down-regulated genes upon THZ1 and JQ1 combination treatment, (excluded when significant effect of the single compounds) (adj.P.Val and log fold change of the combination vs control comparison are depicted)
